# Editorial: Recent advances in endocrinology of non-traditional mammalian models

**DOI:** 10.3389/fendo.2024.1520073

**Published:** 2024-11-18

**Authors:** Daniel William Hart, Nigel C. Bennett, Aaryn Mustoe

**Affiliations:** ^1^ Department of Zoology and Entomology, University of Pretoria, Pretoria, Gauteng, South Africa; ^2^ Mammal Research Institute, University of Pretoria, Pretoria, Gauteng, South Africa; ^3^ Southwest National Primate Research Center, Texas Biomedical Research Institute, San Antonio, TX, United States

**Keywords:** comparative endocrinology, evolutionary endocrinology, hormone-driven adaptations, social structure, environmental resilience

Endocrine research has traditionally relied on common laboratory animals like rats and mice to understand how hormones control vital processes such as growth, metabolism, reproduction, and even social behaviours (1, 2). While these traditional model animals have been incredibly useful, they represent only a small slice of the vast diversity of hormonal systems found in mammals (1, 2). Now, researchers are increasingly turning to non-traditional species—animals that live in unusual environments or have complex social structures (3–6). These species are offering fresh, sometimes surprising insights into how animals adapt and evolve, with exciting implications for biology and medicine alike (3–6).

This Research Topic shines a light on this shift, featuring research on animals like the African mole-rats (Bathyergidae), meerkats (*Suricata suricatta*), and the Southern giant pouched rat (*Cricetomys ansorgei*). These species are helping researchers uncover how hormones enable animals to survive in extreme conditions and navigate intricate social systems.

In the case of the Ansell’s mole-rat (*Fukomys anselli*) these rodents occur underground in low-oxygen environments with scarce food. These cooperative breeders have evolved remarkable adaptations to their harsh habitat, including lower-than-average resting metabolic rates and body temperatures. Despite these low levels of key metabolism-regulating thyroid hormones, like thyroxine (T4), mole-rats have developed the ability to limit the hormone’s effects at the tissue level, consequently conserving energy (Gerhardt et al.). These discoveries may even offer new insights into human conditions with subjects that possess thyroid disorders, showing how research on these lesser-known species can have real medical relevance.

Meerkats, another fascinating group of cooperatively breeding animals, may aid researchers in our understanding of how hormones affect social dynamics and reproduction. Dominant female meerkats produce unusually high levels of androgens, such as testosterone, during pregnancy. This hormonal surge influences the growth and competitiveness of their offspring, but it also comes with trade-offs, such as slower body mass gain and reduced survival rates (Davies et al.). These findings provide valuable lessons about the delicate balance hormones strike between survival and reproductive success—lessons that could even inform how we think about social structures in human societies.

The Southern giant pouched rat is yet another intriguing model for studying how hormones regulate social behaviour. Recent research mapped the distribution of vasopressin and oxytocin receptors in their brains, showing patterns that differ from more common lab animals like hamsters and voles (Freeman et al.). These differences likely reflect the non-model rat’s unique social organization, highlighting the importance of studying diverse species to truly understand hormone-driven behaviour. Similarly, ongoing research on oxytocin, often called the “love hormone,” shows that its role in bonding and social interaction can vary significantly depending on the species and social environment(Sosnowski and Brosnan, 7). This stresses the need for broader comparative research to fully explore the complexities of hormonal systems.

Collectively, these studies show the value of non-traditional mammalian models in expanding the field of endocrinology. While traditional laboratory animals are still important, they cannot capture the full range of hormonal adaptations that exist in nature. By studying species that thrive in extreme environments or possess unique social systems, researchers are uncovering new ways that hormones are involved in animals—and humans— to adapt to life’s challenges. As this exciting field continues to grow, we can expect even more groundbreaking discoveries from these and other remarkable species. The future of endocrine research lies in embracing the diversity of the animal kingdom, unlocking insights that could transform both our understanding of biology and its applications to human health.
